# Correlation of Histopathologic Features with Demographic, Gross and Radiographic Findings in Giant Cell Granulomas of the Jaws

**DOI:** 10.5681/joddd.2013.036

**Published:** 2013-12-18

**Authors:** Amirala Aghbali, Mahmood Sina, Seyyed Mahdi Vahid Pakdel, Parya Emamverdizadeh, Maryam Kouhsoltani, Seyyed Mostafa Mahmoudi, Maryam Janani

**Affiliations:** ^1^Dental and Periodontal Research Center, Tabriz University of Medical Sciences, Tabriz, Iran; ^2^Associate Professor, Department of Oral and Maxillofacial Pathology, Faculty of Dentistry, Tabriz University of Medical Sciences, Tabriz, Iran; ^3^Assistant Professor, Department of Oral and Maxillofacial Pathology, Faculty of Dentistry, Tabriz University of Medical Sciences, Tabriz, Iran; ^4^Students’ Research Committee, Tabriz University of Medical Sciences, Tabriz, Iran; ^5^Post-graduate Student, Department of Prosthodontics, Faculty of Dentistry, Tabriz University of Medical Sciences, Tabriz, Iran; ^6^Assistant Professor, Department of Oral and Maxillofacial Pathology, Faculty of Dentistry, Birjand University of Medical Sciences, Birjand, Iran; ^7^Assistant Professor, Department of Endodontics, Faculty of Dentistry, Tabriz University of Medical Sciences, Tabriz, Iran

**Keywords:** Giant cell, granuloma, histology, radiography

## Abstract

*** Background and aims.*** The correlation between morphology of giant cells in peripheral granulomas of the jaws and the aggressive behavior of the lesion is unknown. This study investigated the correlation between the histopathologic features with demographic, gross and radiographic findings in giant cell granulomas.

***Materials and methods.*** In this analytical study, data from 23 cases of central giant cell granuloma (CGCG) and 42 cases of peripheral giant cell granuloma (PGCG) were analyzed, focusing on age, gender, location, and gross and radiographic features. For each patient, microscopic slides were assessed in terms of histologic features of giant cells and stroma.

*** Results.*** No significant differences were found in the mean number of nuclei or the size of nuclei and giant cell distribution patterns between the jaws and genders in both lesions (P >0.05). Correlation between the mean number of nuclei and age was positively significant and correlation between the size of nuclei and age was negatively significant (P < 0.05). In addition, correlation between the mean number and size of nuclei and the size of the lesion was significant (P < 0.05). Correlation between stroma and aggressiveness of CGCGs was not statistically significant. Correlation between histopathologic features and radiographic findings was not statistically significant (P > 0.05).

***Conclusion.*** There were correlations between the mean number of nuclei per giant cell and the size of the lesion and age, and between the size of nuclei and size of the lesion. No relation was observed between histopathologic and radiographic features.

## Introduction

Central and peripheral giant cell granulomas of the jaw are benign reactive lesions with unknown etiology and pathogenesis.^1^ Histopathologic features of both lesions are characterized by the presence of numerous multinucleated giant cells and mononuclear stromal cells in a fibrous connective tissue.^2^

Central giant cell granulomas (CGCGs) occur within jaw bones and appear as radiolucent defects which may be unilocular or multilocular.^3^ The majority of these lesions are noted in young adults with a predilection for females.^4,5^ There is considerable variation in the clinical behavior. Most of CGCGs (non-aggressive type) are asymptomatic and may be encountered in routine radiographic examinations. Another form of CGCG (aggressive type) is characterized by pain, cortical perforation, root resorption and tendency to recur after treatment.^4,6^

Peripheral giant cell granuloma (PGCG) occurs as a red/purple nodule exclusively on the gingiva and alveolar ridge. These lesions originate from periodontal ligament or mucoperiosteum of the alveolar ridge as a result of local irritation or trauma.^6,7^ PGCG can develop at any age, especially during the fifth and sixth decades of life, with a slight female predilection.^7,8^ In some cases, PGCG affects the underlying bone and may cause cupping resorption.^8^

Differences in biologic and clinical behaviors of GCGs raise the question whether there is a relationship between these behaviors and other features of the lesions such as gross (size and consistency), demographic (age, gender and location) and radiographic findings (cortical perforation and root resorption).

The present study aimed to investigate the correlation of the histopathologic features with demographic, gross and radiographic findings in giant cell granuloma. If such a relationship is established, it can be used for better treatment planning and predicting the prognosis.

## Materials and Methods

### Patients and Inclusion Criteria

The data for this retrospective study were obtained from records of 65 patients with GCG of the jaws (23 CGCG and 42 PGCG), referred to the Department of Oral and Maxillofacial Pathology, Tabriz Faculty of Dentistry, between 2004 and 2009.Inclusion criteria were patients with complete records. In all the cases information relating to age, gender, location, gross (including size and consistency) and radiographic features (including cortical perforation, root resorption and margins) were recorded in checklists.

###  Microscopic Evaluation

**Figure 2.**
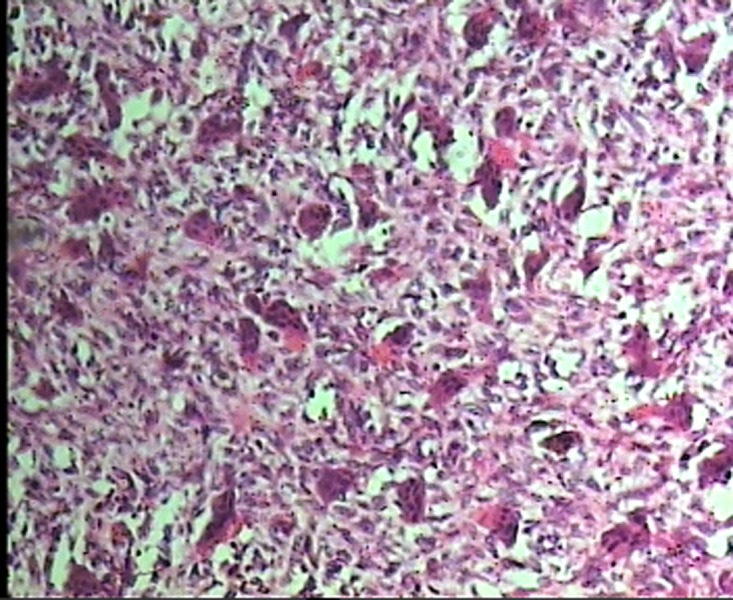
Diffuse distribution of giant cells.

For each patient, microscopic slides were assessed in terms of histologic features of the stroma and giant cells. Two investigators evaluated the specimens without knowledge of the patient’s clinical course. In each case, the histologic examination was performed in 4 random high-power fields (HPF, ×400) under a conventional light microscope (Olympus CH30, Japan). The analyzed parameters included the mean number of nuclei per giant cell, size of nuclei (Figure 1), and distribution pattern of giant cells. The distribution pattern was classified as focal or diffuse (Figure 2). Because histologic features sometimes varied from field to field, the overall and predominant histologic appearance was taken into account for the study.


Figure 1.Different sizes of multinucleated giant cells. A: A large giant cell with numerous nuclei. B: A small giant cells with a few nuclei.

**A:**
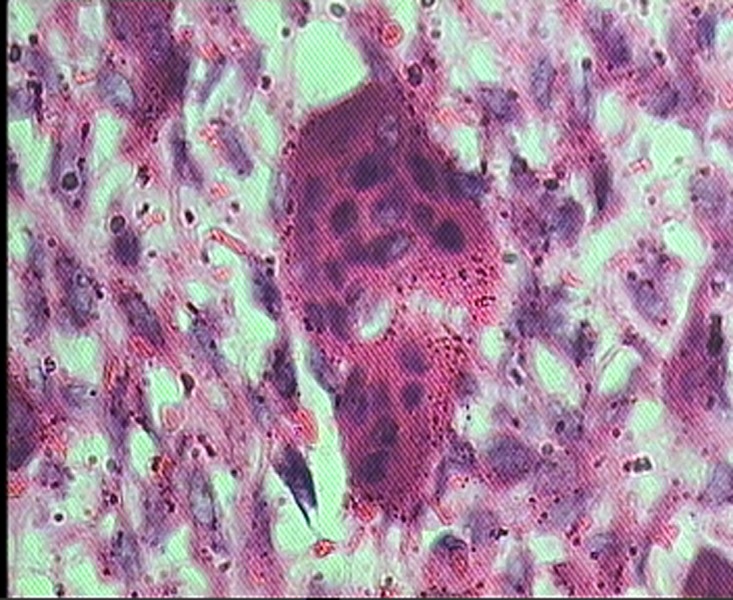

**B:**
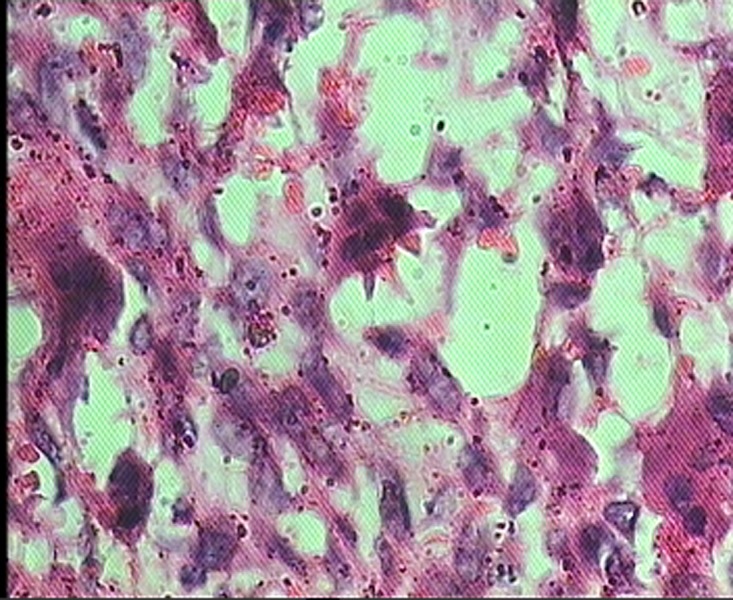

###  Statistical Analysis

Spearman’s rank correlation coefficient analysis was used to correlate histopathologic features with radiographic findings, age, gender, location, size and consistency. Chi-squared test, Student's t-test and Spearman's correlation coefficient were used to compare various group. Statistical significance was defined at P<0.05. SPSS 13.0 (SPSS Inc, Chicago, IL) was used for all the analyses.

## Results

Complete records and tissue specimens from 23 cases of CGCG (15 females and 8 males), and 42 cases of PGCG (24 females and 18 males) were studied. The clinical data for all the reviewed cases, including the age range, mean age, gender information and location of the legions, are listed in Table 1.

**Table 1:** Summary of demographic and anatomic features of cases

	Age	Location	Size
Lesion	Mean (range)	Male/Female	Maxilla/Mandible	(cm)
CGCG	37.5 (10-75)	7/16	16/7	1.84±2.05
PGCG	33.8 (7-70)	19/23	18/24	1.26±1.32

###  Demographic, Anatomic and Radiographic Profile of CGCGs

Among 23 cases of CGCGs, 16 (69.6%) were located in the maxilla and 7 (30.4%) in the mandible. Most lesions were located in the posterior region of the jaws (65%). The mean size of the lesions was 1.84±2.05 cm, and the mean age of the patients was 37.5±23.51 (an age range of 10-75 years). 69.6% of cases of CGCG occurred in women. All the CGCGs demonstrated unilocular radiolucencies. Sixteen (69.6%) lesions presented with well-defined margins and 7 (30.4%) with ill-defined margins. Cortical perforation was found in 18 (78.2%) cases, two (8.6%) lesions revealed evidence of root resorption and one (4.3%) showed tooth displacement.

###  Demographic, Anatomic and Radiographic Profile of PCGCs

Among 42 cases of PGCG, 20 (47.6%) were located in the maxilla and 22 (52.4%) in the mandible. Most lesions were located in the posterior region of the jaws (69%). The mean size of the lesion was 1.26±1.32 cm, and the mean age of the patients was 33.8±17.73 (an age range of 7-70 years). 54.8% of cases of PGCG occurred in women. In 4 (9.52%) cases cupping erosion of the underlying bone was seen.

###  Gross and Histologic Profile of PCGCs and CGCGs

In 86.95% of cases of CGCG and 85.7% of cases of PGCG consistency of the lesion in gross examination was elastic. Mean number of nuclei per giant cell was 9.54±5.73 in CGCGs and 8.58±3.01 in PGCGs, and Student's t-test revealed that the difference was not statistically significant (P=0.250). Size of nuclei was intermediate in 45.2% of CGCGs and 43.5% of PGCGs. Giant cell distribution pattern was diffuse in 87.3% of cases of CGCG and 76.2% of cases of PGCG. Stroma in 39.1% of CGCGs and in 88.1% of PGCGs was hypocellular and fibrillar.

### Correlation of the Histologic and the Demographic Findings 

**Figure 3.**
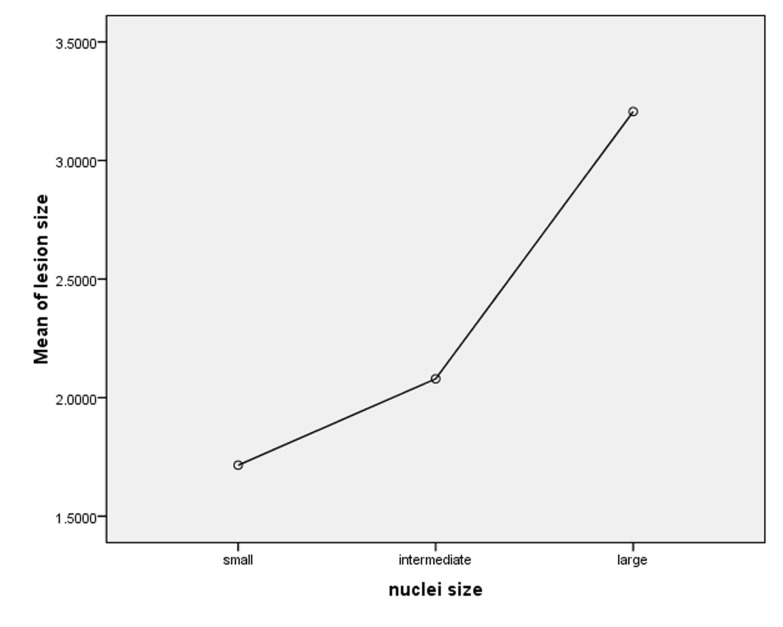
Relation between size of nuclei and size of the lesion.

According to the results of t-test there were no significant differences in the mean number of nuclei or the size of nuclei between the jaws and genders in both lesions (P>0.05). Chi-squared statistics revealed that giant cell distribution patterns between the jaws and genders were not significantly different between the two lesions (P>0.05). Spearman’ scorrelation coefficient indicated that correlation between the mean number of nuclei and age was positively significant and correlation between size of nuclei and age was negatively significant (P<0.05). In addition, correlation between the mean number and size of nuclei and the size of the lesion was significant (P<0.05) (Figure 3). Correlation between stroma and aggressiveness of CGCGs was not statistically significant (P>0.05).

### Correlation of the Radiographic and Histologic Findings 

Spearman’scorrelation coefficient revealed that the correlation between histopathologic features and radiographic findings was not statistically significant (P>0.05).

### Correlation of the Histologic and Gross Findings

No significant difference was found between histopathologic findings and consistency between the two lesions (P>0.05).

## Discussion

‍Central and peripheral giant cell granulomas are common reactive lesions, which occur either peripherally on the gingiva and alveolar ridge or centrally in the bone.^9,10^

Consistent with those of other studies,^6,7^ the results of the present study showed that giant cell granulomas occur more frequently in females. In addition, we found that CGCGs are much more common in women than PGCGs. Our findings showed that PGCGs have a slight predilection for the mandible as reported in previous studies.^1,6^ In the present study, only 30.4% of CGCGs appeared in the mandible, which is in contrast to the proved thesis that describes predilection for the mandible.^1,6^ This discrepancy may be due to specific ethnicity or small sample size.

In radiographic evaluation of aggressive type of CGCGs, no significant correlation was found between cortex perforation and root resorption, as reported by Vered et al and Omallery et al.^11,12^

Microscopic examination of giant cell granulomas shows numerous multinucleated giant cells and mononuclear cells (fibroblast and histiocyte-like cells and monocyte-macrophages) within a prominent fibrous stroma.^1^ The origin and nature of the multinucleated giant cells has been a subject of debate.^13^ Some giant cells may contain a few small nuclei and others may demonstrate large, numerous nuclei.^14^

In this study 30.4 % of cases of CGCG and 21.4% of cases of PGCGs demonstrated large nuclei and more than 40% of cases in both lesions showed intermediate nuclei. Mean number of the nuclei per giant cell in CGCGs and PGCGs exhibited statistically significant correlation with the size of the lesion and age. Giant cells may distribute focally or diffusely in the stroma,^6^ but in our study 87.3% of CGCGs and 76.2% of PGCGs had a diffuse pattern. Therefore, the present study proves that giant cells may mostly be distributed diffusely.

In this study we also investigated correlation between stroma and radiographic features of giant cell granulomas; 39.1% of CGCGs and 88.1% of PGCGs were hypocellular and fibrillar. According to the data in the present study the stroma in PGCGs is frequently hypocellular and fibrillar.

## Conclusion

The above findings indicate that as the lesion size increases the size and number of nuclei in giant cells increase. No relation was observed between histopathologic and radiographic features in aggressive and non-aggressive types of CGCG. A prospective study with more samples may be useful in evaluating whether the histopathologic findings can be used to plan better treatment and predict prognosis.
